# Longstanding Endobronchial Foreign Body

**DOI:** 10.1155/DTE.5.257

**Published:** 1999

**Authors:** R. Trisolini, R. Dore, R. Bertolini, L. Pasturenzi, A. Fede Catania, G. Gualtieri, M. Luisetti, M. Torre

**Affiliations:** 1 Clinica di Malattie dell'Apparato Respiratorio Università degli Studi di Pavia IRCCS Policlinico S. Matteo via Taramelli 5 Pavia 27100 Italy; 2 Istituto di Radiologia Università degli Studi di Pavia IRCCS Policlinico S. Matteo via Taramelli 5 Pavia 27100 Italy; 3 Centro A. de Gasperis Divisione di Chirurgia Toracica Ospedale Niguard Cà Granda Milano 20131 Italy

## Abstract

There are many circumstances in which the diagnosis of endobronchial inhalation of a foreign body (FB) can be missed. Generally, in such cases, within weeks or at most months from the event, clinical bronchopulmonary symptoms develop which allow a correct diagnosis to be made and significant complications to be avoided. We report the case of a patient in whom an endobronchial FB remained undiagnosed, because of lack of symptoms, for almost three years, and then caused signifiicant complications before being identified and removed. Problems related to diagnosis and therapy are discussed.


*Diagnostic and Therapeutic Endoscopy*, Vol. 5, pp. 257–261
Reprints available directly from the publisher
Photocopying permitted by license only

© 1999 OPA (Overseas Publishers Association) N.V.
Published by license under
the Harwood Academic Publishers imprint,
part of The Gordon and Breach Publishing Group.
Printed in Malaysia.

### Longstanding Endobronchial Foreign Body: A Case Report

R. TRISOLINI[a], R. DORE[b], R. BERTOLINI[a], L. PASTURENZI[a], A. FEDE CATANIA[a], G. GUALTIERI[a], M. LUISETTI[a,*] and M. TORRE[c]

[a]*Clinica di Malattie dell'Apparato Respiratorio,* [b]*Istituto di Radiologia, Università degli Studi di Pavia, IRCCS Policlinico S. Matteo, via Taramelli 5, 27100 Pavia, Italy,* [c]*Centro A. de Gasperis, Divisione di Chirurgia Toracica, Ospedale Niguarda Cà Granda, 20131 Milano, Italy*

*(Received 4 November 1998; Revised 26 January 1999; In final form 2 March 1999)*

**There are many circumstances in which the diagnosis of endobronchial inhalation of a foreign body (FB) can be missed. Generally, in such cases, within weeks or at most months from the event, clinical bronchopulmonary symptoms develop which allow a correct diagnosis to be made and significant complications to be avoided. We report the case of a patient in whom an endobronchial FB remained undiagnosed, because of lack of symptoms, for almost three years, and then caused significant complications before being identified and removed. Problems related to diagnosis and therapy are discussed.**

*Keywords:* Endobronchial foreign body, Fiberoptic bronchoscopy, Pneumonia, Spiral CT scan

### INTRODUCTION

Aspiration of a foreign body (FB) is a rare event in adults, particularly in the absence of predisposing conditions such as deglutition disorders, neuromuscular disorders or alcohol or sedatives abuse, although it is quite frequent and dangerous in children (asphyxia caused by the presence of FBs in the airways is the 4th most common cause of death in children between the ages 1–4) [1–3]. What is more, foreign bodies in the adult are usually identified and removed promptly. Here we report the case of a 56 year old patient who had an endobronchial foreign body, asymptomatic and undiagnosed for about 33 months before the abrupt onset of symptoms, following which the FB was diagnosed and removed.

### CASE REPORT

A male, non-smoking, 56 year old patient was referred to our department in April 1997 with a three day history of fever, pain at the base of the right lung, dyspnea and a productive cough. His past

* Corresponding author. Tel.: (+39)0382-423131. Fax: (+39)0382-502269. E-mail: luisetti@telnetwork.it.

clinical history included a right sided pneumonia in 1972 (he suffered afterward from frequent episodes of bronchitis) and an acute cerebrovascular accident in 1994.

On admission his blood pressure was 120/90 mmHg and heart rate was 120 beats $\cdot$ $min^{-1}$. Physical examination revealed a total absence of breath sounds in the middle and basal right lung fields and a relevant hepatomegaly. A chest X-ray showed an abundant pleural effusion on the right side and an increased heart shadow, in particular in the region of the left ventricle. Electrocardiography (ECG) was normal. Arterial blood gas values breathing under oxygen supplementation at the flow of 2 l $\cdot$ $min^{-1}$ (prescribed by the emergency room of our hospital) were: $PaO_2$ 73 mmHg; $PaCO_2$ 26 mmHg; pH 7.43. The blood count showed an increase in white blood cells (WBC = 15,330/$mm^3$) and in particular in neutrophils (88%).

Despite a week of antibiotic treatment and a thoracentesis, the radiological examination did not show any improvement, so we performed a fiber-optic bronchoscopy which revealed the presence of a white object, surrounded by granulation tissue, almost completely obstructing the intermediate bronchus (Fig. 1). When specifically asked, his wife remembered that the patient could have swallowed the cap of the intravenous catheter that had been used during his home convalescence after the cerebrovascular accident in 1994; after which, he had a mild non-productive cough for a short period of time. Spiral computed tomography (CT) of the chest, performed after the endoscopy, confirmed the presence of a foreign body and showed retraction and fibrosis of the lower and middle lobes of the lung parenchyma (Fig. 2(a) and (b)). The middle lobe also had bronchiectasis.

The patient underwent a new course of antibiotic therapy with a significant improvement of the clinical picture (remission of the fever, dyspnea and chest pain) and of the arterial blood gas values, while the CT findings remained substantially the same. At this point, steps were taken to remove the FB with a rigid bronchoscope after a failed attempt with the fiberoptic bronchoscope.

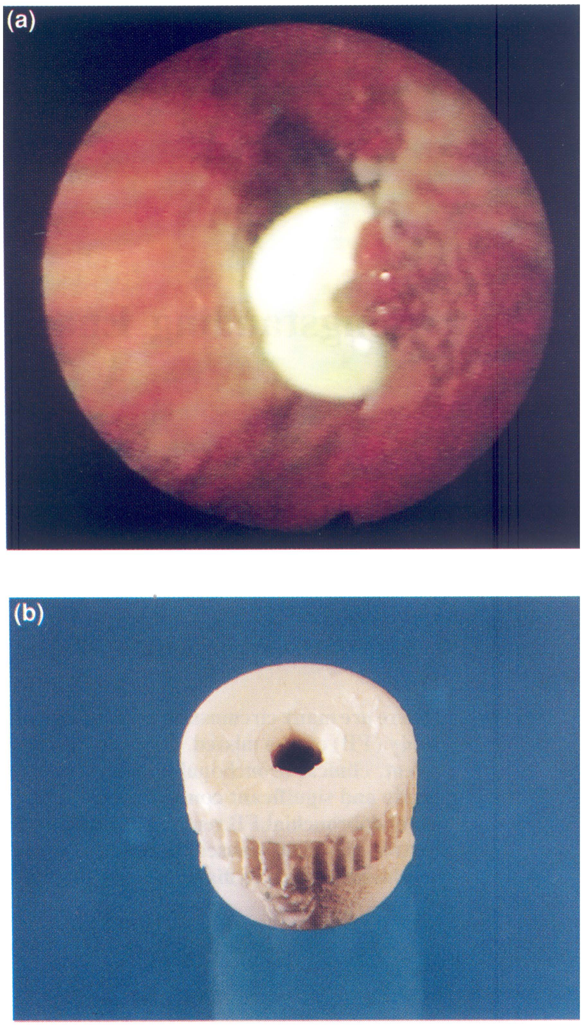

FIGURE 1 (a) Fiberoptic bronchoscopy finding of the white object obstructing the intermediate bronchus. (b) The object after removal.

### DISCUSSION

Adult patients who have aspirated an FB can be divided into two groups (acute and chronic) based upon the amount of time between aspiration and diagnosis [4]. The indications for performing endoscopy differ between the two groups.

According to the most comprehensive studies, the main indications for diagnostic bronchoscopy in patients in the "acute group" (retention of an FB for

(a)

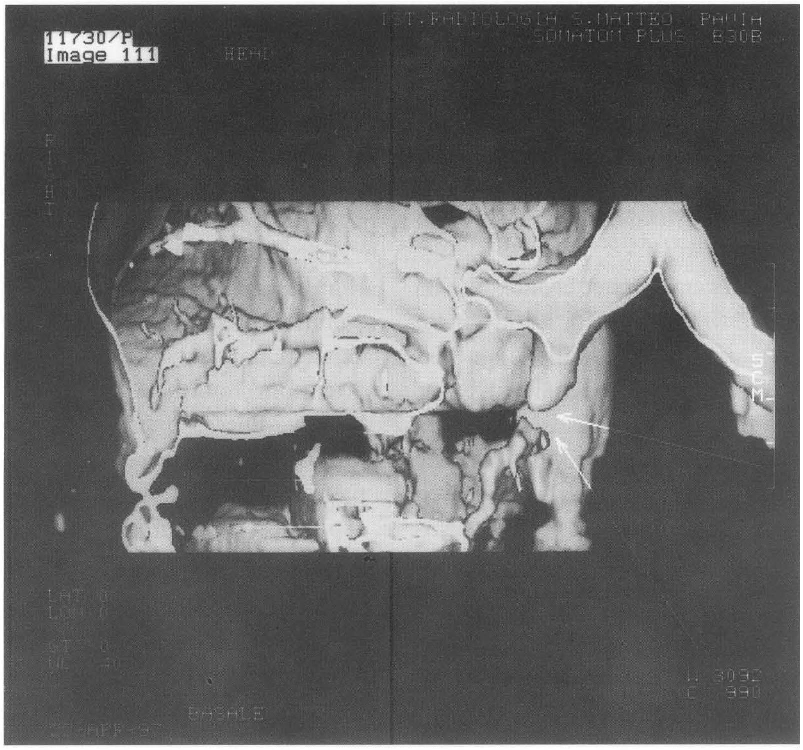

(b)

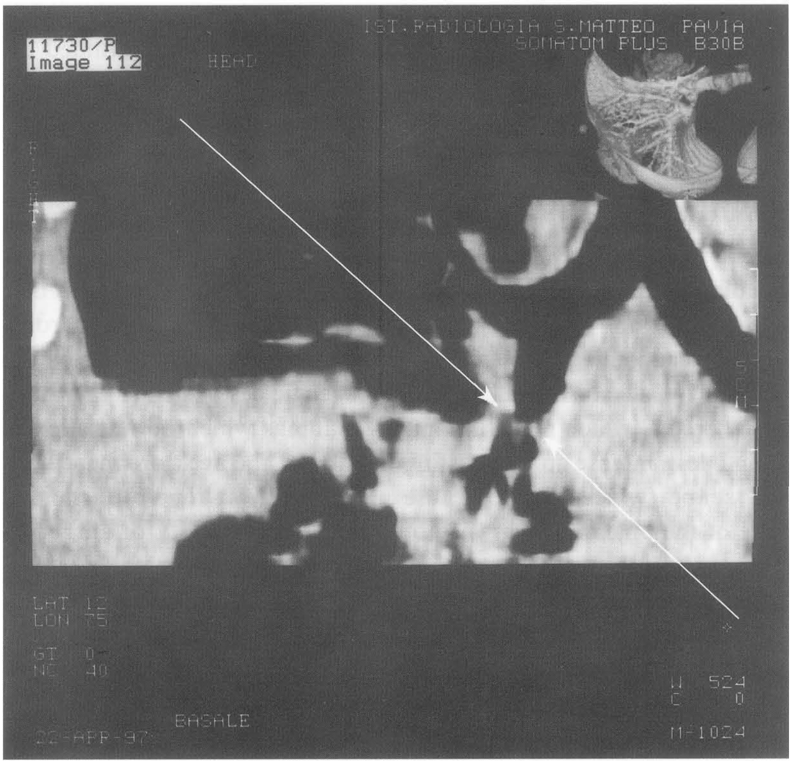

FIGURE 2 (a) 3D appearance of right bronchial system: the main, the upper, the stenotic intermediate and the lower bronchi are easily discernible. (b) Paracarinal section corresponding to Fig. 2(a): arrows show the low density membrane occluding the intermediate bronchus.

not more than one week) are a positive history and the presence of a visible object on the chest X-ray, whereas the main indications in the "chronic group" (retention for more than one month) are a positive history of swallowing an FB and the suspicion of an endobronchial lesion.

Most scientific articles on tracheobronchial FBs in adults are concerned with acute aspiration which, being generally symptomatic, are rapidly diagnosed and removed. Even when aspiration results in few or no symptoms and an early diagnosis is not made, there are often, in the first weeks or months after the event, clinical symptoms caused by early or delayed complications [4–8]; these symptoms are investigated by a series of examinations which lead to the identification and then removal of the FB [9–11].

The peculiarity of the case we report is that the acute aspiration, causing few (or even no) symptoms, was followed by a healthy period of 33 months; in fact examining the clinical history of the patient we see that, from the time of the event until the diagnosis, he suffered only a few bouts of bronchitis, not dissimilar to the ones he had experienced in the years before the aspiration and, therefore, not definitely attributable to the presence of the FB.

The initial unimportance given to any symptoms can be linked to the compromised neurological condition of the patient, who was convalescing after an acute cerebrovascular accident, while the lack of symptoms and/or complications for such a long period of time after the event could be explained, as was already hypothesised by Jackson in 1921 [12], by the inert nature of the object (which made inflammation less likely and less intense in the resting place); the rounded shape (which did not cause trauma), and the size of the object (which was not large enough to obstruct the intermediate bronchus completely). Another important factor was the absence of chronic pulmonary diseases, which would have already limited respiratory function by the time of the event.

The first point of reflection offered by this case is an epidemiological one. The ever-increasing use of invasive diagnostic and therapeutic methods is paralleled by an increased risk of "iatrogenic aspirations". In fact, in some large surveys, dental or medical appliances account for a substantial number of aspirated FBs, and our case is but one example.

Furthermore, we believe that bronchopulmonary changes, particularly in the presence of the more common predisposing situations, should make the clinician suspicious of FB aspiration. Particular attention should be given to taking the patient's history. Direct questioning about the possibility of having "swallowed" an object is necessary since patients and their families often do not mention the aspiration of an object because they do not realise its importance.

From the clinico-radiological point of view greatest attention should be paid to a suspicious endobronchial lesion: in fact, in most cases with a negative history a diagnostic bronchoscopy is performed to investigate conditions such as: recurrent pneumonia in the same area, delayed resolution pneumonia, pneumonia with lung volume loss and pneumonia with continued fever despite adequate treatment.

The treatment of choice is the endoscopic removal of the FB, which is effeotive in around 98% of cases. Although the rigid bronchoscope must today be considered the safest and most effective instrument for removing an FB, in recent years more and more studies have demonstrated that good results can be obtained with fiberoptic bronchoscopes, which can be of great importance in avoiding surgery in specific cases such as the presence of cervico-facial traumas which do not allow effective hyperextension of the neck or when the FB is too distal to be reached by a rigid bronchoscope [13,14].

The possibility of surgery, which is quite rare, must be considered carefully case by case and limited to these three types of situation: FBs not removable by endoscopy due to their size and/or shape; FBs which have been *in situ* for a long time and which have provoked pathologic changes in the tissue beneath the obstruction; small objects which have reached the periphery and are impossible to reach or extract by endoscopy [15].

In conclusion, when a patient presents with an endobronchial lesion of unknown origin, particularly if associated with predisposing factors, the wise clinician should question the patient directly about the possibility of FB aspiration and not discount its relevance even if many months antecedent to the onset of the patient's symptoms. Fiberoptic bronchoscopy is usually diagnostic and can also often be successfully used to remove the FB.

### *References*

[1] McGuirt, W.F. *et al.* Tracheobronchial foreign bodies. *Laryngol.* 1988; **98**: 615–618.

[2] Banerjee, A. *et al.* Laringo-tracheobronchial foreign bodies in children. *J. Laryngol. Otol.* 1988; **102**: 1029–1032.

[3] Mantel, K. and Butenandt, I. Tracheobronchial foreign body aspiration in childhood. A report of 224 cases. *Eur. J. Pediatr.* 1986; **145**: 211–216.

[4] Lan, R.S. Non-asphyxiating tracheobronchial foreign body in adults. *Eur. Resp. J.* 1994; **7**(3): 510–514.

[5] Jackson, C. Symptomatology and diagnosis of foreign bodies in the air and food passages. *Am. J. M. Sc.* 1921; **161**: 625.

[6] Jackson, C. and Jackson, C.L. *Diseases of the Air and Food Passages of Foreign Body Origin.* Philadelphia: W.B. Saunders Co, 1936.

[7] Limper, A.H. and Prakash, U.B.S. Tracheobronchial foreign bodies in adults. *Ann. Int. Med.* 1990; **112**: 604–609.

[8] Linton, J.S. Longstanding tracheobronchial foreign bodies. *Thorax* 1957; **12**: 164–170.

[9] Erren, J.P. and Shipmann, R. Recurrent retention pneumonia on right with changing site after aspiration of dental impression material with almost complete casting of a segmental bronchus. *Pneumologie* 1995; **49**(11): 601–603.

[10] Weissberg, D. and Schwartz, I. Foreign bodies in the tracheobronchial tree. *Chest* 1987; **91**: 730–733.

[11] Wolkove, N. *et al.* Occult foreign-body aspiration in adults. *JAMA* 1982; **248**: 1350–1352.

[12] Jackson, C. Prognosis of foreign body in the lung. *JAMA* 1921; **77**: 1178–1182.

[13] Zavala, D.C. and Rhodes, M.L. Experimental removal of foreign body by fiberoptic bronchoscopy. *Am. Rev. Res. Dis.* 1976; **110**: 357–360.

[14] Hiller, C. *et al.* Foreign body removal with the flexible fiberoptic bronchoscopy. *Endoscopy* 1977; **9**: 216–222.

[15] Buonsanto, A. *et al.* Endobronchial foreign bodies: surgical indications. *Minerva Chir.* 1996; **51**: 997–1003.

